# Results of lumbar transforaminal epidural steroid injections based on the physician referral source

**DOI:** 10.1016/j.inpm.2022.100114

**Published:** 2022-07-31

**Authors:** Josh Levin, Wei-Xian Li, Nolan Gall, John Chan, Marc Caragea, Lisa Huynh, Jayme Koltsov, Matt Smuck

**Affiliations:** a Department of Orthopaedic Surgery, Stanford University, 450 Broadway St., Pavilion C, 4th Floor, MC 6342, Redwood City, CA, 94063, USA; b Department of Neurosurgery, Stanford University, 450 Broadway St., Pavilion C, 4th Floor, MC 6342, Redwood City, CA, 94063, USA; c Division of Physical Medicine and Rehabilitation, University of Utah, 30 N 1900 E, Salt Lake City, UT, 84132, USA

## Abstract

**Background:**

Many patients who receive lumbosacral transforaminal epidural steroid injections (TFESIs) are referred for the injection from a physician who does not perform the procedure.

**Purpose:**

To compare success rates of fluoroscopically guided lumbosacral TFESIs in patients who had a clinical evaluation and recommendation for the injection by a fellowship-trained spine specialist who routinely performs ESIs (Group A), vs those who had a clinical evaluation by a fellowship-trained spine specialist who referred the patient for the procedure to be done by a different physician (Group B).

**Study design/setting:**

Retrospective, observational, in vivo study of consecutive patients. Patient.

**Sample:**

Patients undergoing lumbosacral TFESIs at a single outpatient academic spine center. Outcome.

**Measures:**

Numeric Rating Scale (NRS) pain score improvement.

**Methods:**

Current procedural terminology (CPT) codes were used to search all consecutive patients who received a lumbosacral TFESI between September 2019 and September 2020. All patients with pre- and post-injection NRS pain scores within 60 days of the injection were included in the analysis.

**Results:**

A total of 230 TFESIs were analyzed, 151 in Group A, and 79 in Group B. The primary outcome was defined as > 50% improvement in the NRS pain score. 39% [95% CI: 33, 45%] of all patients who received a lumbosacral TFESI achieved a successful outcome. There were better results in Group A with a 47% [95% CI: 39, 55%] success rate compared to a 23% [95% CI: 14, 32%] success rate in Group B. Group A also had a higher proportion of patients who achieved at least 80% pain relief (26% [95% CI: 19, 33%]) compared to Group B (10% [95% CI: 3, 17%]). History of prior surgery did not significantly affect outcomes.

**Conclusion:**

This retrospective study demonstrated a higher success rate from lumbosacral TFESIs when patients were referred for the injection by a physician who performs ESIs.

## 1 Introduction

Physicians from multiple specialties perform epidural steroid injections (ESIs), most commonly anesthesiologists and physiatrists [1], with fewer done by radiologists, orthopedic surgeons, neurosurgeons, general surgeons, neurologists, psychiatrists, and others [2,3]. ESIs are often ordered by a physician who does not perform the injection. We recently showed a higher success rate in patients who were referred for a cervical ESI by a spine specialist who routinely performs these procedures, compared to those who were referred for the injection by a spine specialist who does not perform them [4].

Lumbosacral transforaminal epidural steroid injections (TFESIs) are target-specific injections that deliver medication to a specific nerve root(s) for the treatment of radicular pain. Prior to addressing the procedural and safety nuances for the proper execution of a lumbosacral TFESI, practitioners must decide if the patient is a good candidate for the TFESI based on the history, physical exam, and imaging findings. The practitioner will then plan a specific approach for the injection, based on the patient's site of pathology and particular anatomy, and the anticipated flow of injected medication from the selected needle location [5]. Thus, it is possible that the outcomes from target specific lumbosacral TFESIs are influenced by the training and experience of the ordering provider.

This study was conceived to examine differences in lumbosacral TFESI outcomes from TFESIs that are ordered by spine specialists who commonly perform these procedures compared to TFESIs ordered by spine specialists who do not perform them. Since a history of previous lumbosacral spine surgery is known to reduce the success rate of subsequent spine surgery [[6], [7], [8], [9], [10], [11]] the influence of a history of prior lumbosacral spine surgery will also be examined.

## 2 Materials/methods

This study was conducted according to the Declaration of Helsinki with Institutional Review Board (IRB) approval (IRB#48537) obtained from our academic medical center for a single site retrospective observational study. The methods for this study are similar to that described in our prior work [4]. In brief, Current Procedural Terminology (CPT) code 64483 (lumbosacral transforaminal epidural injection) was used to search the electronic medical record for eligible patients who received a lumbosacral TFESI from any of the institution's three fellowship-trained interventional spine physiatrists – all practicing according to Spine Intervention Society Guidelines [12].

A chart review was performed to find all consecutive patients, aged ≥18 years, who underwent a lumbosacral TFESI between September 1, 2019, and September 1, 2020. Exclusion criteria included patients without numerical rating scale (NRS) pain scores in the medical record both before and within 60 days after the TFESI, patients with a baseline NRS score below 4/10, patients who received a diagnostic injection without steroid, and patients who underwent non-epidural injections (i.e., greater trochanter injections, intra-articular hip injections, etc.) on the same day as the lumbosacral TFESI.

Prior to the TFESI, patients had a clinic evaluation with the next available fellowship-trained spine specialist (spine physiatrist or spine surgeon), regardless of subspecialty unless a preference was specified by the patient or referring provider. The spine specialist then ordered the TFESI to be performed by one of the three spine physiatrists. Patients who were referred by one of the physiatrists were generally, but not always scheduled with that same physiatrist. When a TFESI was ordered by one of the spine surgeons, the physiatrist reviewed the request for the injection and made the final decision about the segmental level and side of the injection during a pre-procedure evaluation of the patient on the day of the injection. All injections were performed under fluoroscopic guidance following the Spine Intervention Society Practice Guidelines [12]. Dexamethasone was used for all injections.

### 2.1 Theory

We hypothesize that patients referred from spine specialists who routinely perform lumbosacral TFESIs will have higher rates of success than patients referred from spine specialists who do not perform these procedures.

### 2.2 Statistical analysis

Success in the primary outcome was defined as a Numeric Rating Scale (NRS) pain score improvement of ≥50% from pre-to post-injection. The proportion of patients who achieved this favorable response was calculated using baseline and follow-up encounter NRS pain scores, and Wald 95% confidence intervals were calculated. Success rates were compared between patients who were evaluated and referred for the injection by a spine specialist who performs these procedures (Group A) and patients who were evaluated and referred by a spine specialist who does not performs these procedures (Group B). Similar calculations were performed for patients who achieved ≥80% pain relief.

Univariate differences between those in Group A vs. Group B were conducted with chi-squared and Fisher's exact tests for categorical variables, and Mann Whitney U tests for continuous variables. When necessary, post-hoc tests were conducted pairwise and corrected for multiple comparisons with the Bonferroni method. To verify that NRS scores changed from pre-to post-injection within each group, Wilcoxon rank-sum tests were used. A subgroup analysis was performed based on history of previous surgery (no surgery, previous non-fusion surgery, and previous fusion). These univariate tests were conducted as above and were corrected for multiple comparisons with the Bonferroni method to account for the tests being run in the three subgroups. Additionally, to assess whether there were differences in pain improvement after adjusting for the differences in surgical history (none, prior non-fusion surgery, prior fusion surgery) between groups, multivariable binary logistic regression analyses were used for change (≥50% and ≥80% improvement in NRS), and a similar multivariable generalized estimating equation (GEE) was used to assess the raw change in NRS scores. These models included group, surgical history, and their interaction in order to determine if the change between groups differed among patients with different surgical histories. If not significant or trending, the interaction term was dropped from these models. All analyses were performed with SAS version 9.4 (Cary, NC, USA) with a two-sided level of significance of 0.05.

## 3 Results

Overall, 369 consecutive patients met the search criteria. A total of 139 patients were excluded due to lack of follow-up NRS data within 60 days (108 patients), baseline NRS pain score <4/10 (21 patients), receiving additional injections on the same day as the lumbosacral TFESI (8 patients), or aborted procedures due to pain (2 patients). A total of 230 patients remained and were included in the analysis, 151 in Group A and 79 in Group B. Sixty-eight of the 79 patients in Group B were referred by one of 4 fellowship-trained orthopedic spine surgeons, 9 patients were referred by one of 4 fellowship-trained spine neurosurgeons, and 2 patients were referred by orthopedic joint surgeons.

There were no significant differences between groups in age (p ​= ​0.816), gender (p ​= ​0.658), baseline NRS pain scores (p ​= ​0.963), and time to follow-up (p ​= ​0.318) (Table 1). Group B had more patients with a history of prior non-fusion lumbar spine surgery (p ​< ​0.001), and more patients with a history of prior lumbar fusion (p ​< ​0.001) (Table 1).

**Table 1:** Baseline demographic and clinical information, presented as n (%) for categorical variables with 95% confidence intervals, and median [interquartile range (IQR)] unless otherwise noted for continuous variables.

	Group A (referred by physician who performs ESIs)	Group B (referred by physician who does not perform ESIs)	p-value
Age (mean [95% CI] years)	69 [54, 77]	69 [58, 75]	0.816
Female	83 (55%)	41 (52%)	0.658
Baseline NRS pain score	6 [5,8]	7 [5,8]	0.963
Time to follow-up (days)	20 [14, 28]	20 [14, 35]	0.318
Surgical History:			<0.001∗
None	127 (84%)	34 (43%)	
Non-fusion surgery	12 (8%)	21 (27%)	
Fusion surgery	12 (8%)	24 (30%)	

ESIs ​= ​epidural steroid injections; 95% CI ​= ​95% confidence interval.

∗p ​< ​0.05.

Injections were performed at all levels of the lumbosacral spine from L1 to S1, with the highest proportion of injections performed at L5-S1, followed by L4-5. The only level with a statistically significant difference between the two groups was L2-3, with a larger proportion in Group B having an injection performed at this level compared to Group A. Of the 230 patients, there were a total of 299 levels injected (46 patients in Group A, and 23 in Group B had either bilateral 1-level injections or unilateral 2-level injections; all others were unilateral 1-level injections) (Table 2).

**Table 2:** Number of injections at each spinal level presented as n (%).

	Group A (referred by physician who performs ESIs)	Group B (referred by physician who does not perform ESIs)
L1-2	1 (1%)	4 (4%)
L2-3	2 (1%)	10 (10%)
L3-4	19 (10%)	18 (18%)
L4-5	48 (25%)	27 (26%)
L5-S1	100 (51%)	41 (39%)
S1-2	25 (13%)	4 (4%)

ESIs ​= ​epidural steroid injections; 95% CI ​= ​95% confidence interval.

For the primary outcome of ≥50% reduction in NRS pain score, the overall success rate for all subjects was 39% [95% CI: 33, 45%]. Group A achieved statistically significantly better results with a 47% [95% CI: 39, 55%] success rate compared to a 23% [95% CI: 14, 32%] success rate in Group B. Group A also had statistically significantly greater success in achieving the secondary outcome of ≥80% reduction in NRS pain score with 26% [95% CI: 19, 33%] success compared to 10% [95% CI: 3, 17%] success in Group B (Table 3).

**Table 3:** Patients with a successful outcome from lumbosacral TFESI. Displayed as n, % [95% CI].

	Group A (referred by physician who performs ESIs)	Group B (referred by physician who does not perform ESIs)	p-value
≥50% reduction in NRS	71, 47% [95% CI: 39, 55%]	18, 23% [95% CI: 14, 32%]	p ​< ​0.001∗
≥80% reduction in NRS	39, 26% [95% CI: 19, 33%]	8, 10% [95% CI: 3, 17%]	P ​< ​0.001∗

ESIs ​= ​epidural steroid injections; NRS ​= ​numeric rating scale; 95% CI ​= ​95% confidence interval.

∗p ​< ​0.05.

Median NRS pain scores improved for both groups from pre-to post-injection, from 6 [IQR: 5, 8] to 4 [IQR: 1, 6] in Group A, and from 7 [IQR 5, 8] to 5 [IQR 4, 7] in Group B (p ​< ​0.001 for both). Overall, group A experienced a greater improvement in pain compared to Group B (p ​< ​0.001) along with a lower average pain score post-injection (p ​< ​0.001).

The subgroup analysis based on previous surgical history was underpowered for patients who had undergone prior non-fusion surgery and for those who had undergone prior lumbar fusion. The subgroup analysis remained underpowered when combining patients with any previous surgery into one group. Regardless of prior surgical history, patients in Group A and Group B both demonstrated statistically significant improvements in median NRS pain scores from pre-to post-injection. In patients without a history of prior lumbar spine surgery, those in Group A had significantly greater success (≥50% improvement in NRS) compared to Group B (47% [95%CI: 38, 56%] vs 21% [95%CI: 7, 35%]; p ​= ​0.015). The raw change in NRS score was also greater for Group A, however this was only trending after Bonferroni correction (2.5 [IQR: 0, 5] vs 0 [IQR: 0, 3], p ​= ​0.074). Additionally, in patients with a history of prior lumbar fusion, there was a trend towards better results for patients in Group A in achieving at least 80% pain relief (42% vs. 8%, p ​= ​0.087 after Bonferroni correction). Otherwise, there were not significant differences between Groups A and B in the subgroup analysis.

The multivariable models showed that the pre-to post-injection improvements in pain did not differ among the previous surgery groups (no prior surgery, prior non-fusion surgery, and prior fusion) (p-values for interaction term ​= ​0.871 for ≥50% change, ​= ​0.389 for ≥80% change, and ​= ​0.855 for the raw change). After adjusting for previous surgery, the differences in pain improvement remained significant between groups. The odds for a ≥50% improvement were 3.2 (95% CI 1.6, 6.4) (p ​= ​0.001) for Group A vs Group B, and the odds for a ≥80% improvement were 3.4 (95% CI 1.5, 8.9) (p ​= ​0.007) for Group A vs Group B. After adjustment for previous surgery, the improvement in raw pain scores were 1.3 points (0.5, 2.1) higher in Group A relative to Group B (p ​= ​0.001).

## 4 Discussion

Similar to our study on cervical ESIs [4], we showed a higher success rate from lumbosacral TFESIs when patients were referred for the injection by a spine specialist who routinely performs these procedures. These findings further support the idea that physicians who perform a procedure may be more suited to select patients for that procedure. More importantly, they may better recognize the limitations of the procedure and recommend against it for those unlikely to benefit.

Commonly, patients are referred for ESIs by physicians who do not perform the injections. While there are multiple ways to manage this, at our center, during the timeframe of this study, patients arrived on the day of the procedure with the expectation to receive the scheduled injection. They were evaluated in the pre-procedure suite to make a final decision about whether to perform the requested injection or not. Most injections were confirmed and proceeded as requested, however sometimes the level or approach was changed in response to clinical or radiographic correlation. It was uncommon in this scenario to cancel the injection. We suspect that the more subtle decision about which approach to perform or which level to inject, while important, is less important than the larger decision about whether a percutaneous intervention is the optimal treatment for this patient at that time. We think that a more comprehensive outpatient clinic visit, as opposed to a brief evaluation in a pre-procedure suite, is a better environment to conduct the shared decision making around the various treatment options, and to set treatment expectations. Ultimately, we suspect that our in-clinic evaluations lead to better epidural steroid injection outcomes by excluding some patients who are less likely to benefit from an injection, and by setting more appropriate patient expectations regarding the injection.

Since surgical outcomes have been shown to be worse in patients who have previously undergone spine surgery, we looked to see if a history of spine surgery would affect outcomes from ESIs. With the sample size available, we found no evidence that the difference in outcomes between patients referred from a spine specialist who routinely performs ESIs and those referred from a spine specialist who does not perform ESIs differed based on surgical history.

Strengths of our study include a large patient sample of prospectively collected data. Multiple physicians evaluated the patients, including three fellowship-trained interventional spine physiatrists and ten surgeons, thereby minimizing the biases that can be observed from patients seen by a smaller number of physicians.

Our study is limited by its retrospective nature and the biases associated with this study design. Baseline differences existed between the two groups, with larger proportions of patients in Group B having a history of prior lumbosacral spine surgery. Yet, while previous studies have shown poorer results for surgery in patients with prior surgery [8], we did not see significant differences in outcomes with ESIs when adjusting for history of prior surgery.

Another limitation to our study is that follow-up patterns could have differed between the two groups. For example, it is possible that patients with successful outcomes who were referred from surgeons were less likely to follow-up compared to patients with successful outcomes who were referred from physiatrists. Additionally, differences in expectations about the injections could have existed favoring successful outcomes in patients referred by the physicians who perform the injections. Other than the baseline differences in surgical history between the two groups, we suspect that baseline patient characteristics were similar between the two groups, demonstrated by similar baseline median pain scores and patient demographics. Lastly, our study was limited to the evaluation of NRS pain scores, so no information about functional status was obtained.

## 5 Conclusion

Our retrospective analysis demonstrated a higher success rate from lumbar TFESIs in patients referred for the injection from a spine specialist who routinely performs these procedures, compared to patients referred for the injection from a spine specialist who does not. A history of prior lumbosacral spine surgery did not alter this outcome.

## Declaration of interests

The authors declare the following financial interests/personal relationships which may be considered as potential competing interests: Josh Levin reports a relationship with Scilex Pharmaceuticals that includes: consulting or advisory. Matthew Smuck reports a relationship with Sollis Therapeutics that includes: consulting or advisory. Matthew Smuck reports a relationship with Spine Biopharma that includes: board membership and consulting or advisory. Matthew Smuck reports a relationship with Relievant Medsystems that includes: funding grants.
